# Supplementary material for: Splice-Junction-Based Mapping of Alternative Isoforms in the Human Proteome
Source: Cell Rep. Author manuscript; Available in PMC 2020 Jan 15. (PMC6961840; doi:10.1016/j.celrep.2019.11.026)
Supplement: 2 [file NIHMS1546469-supplement-2.zip › DF1/PXD006675/RightCoronaryArtery/RightCoronaryArtery_8_CCS_GSGQLRVQDVEVHLEDQMVLVHTTLPSQEVQALLEGTGR.pdf]

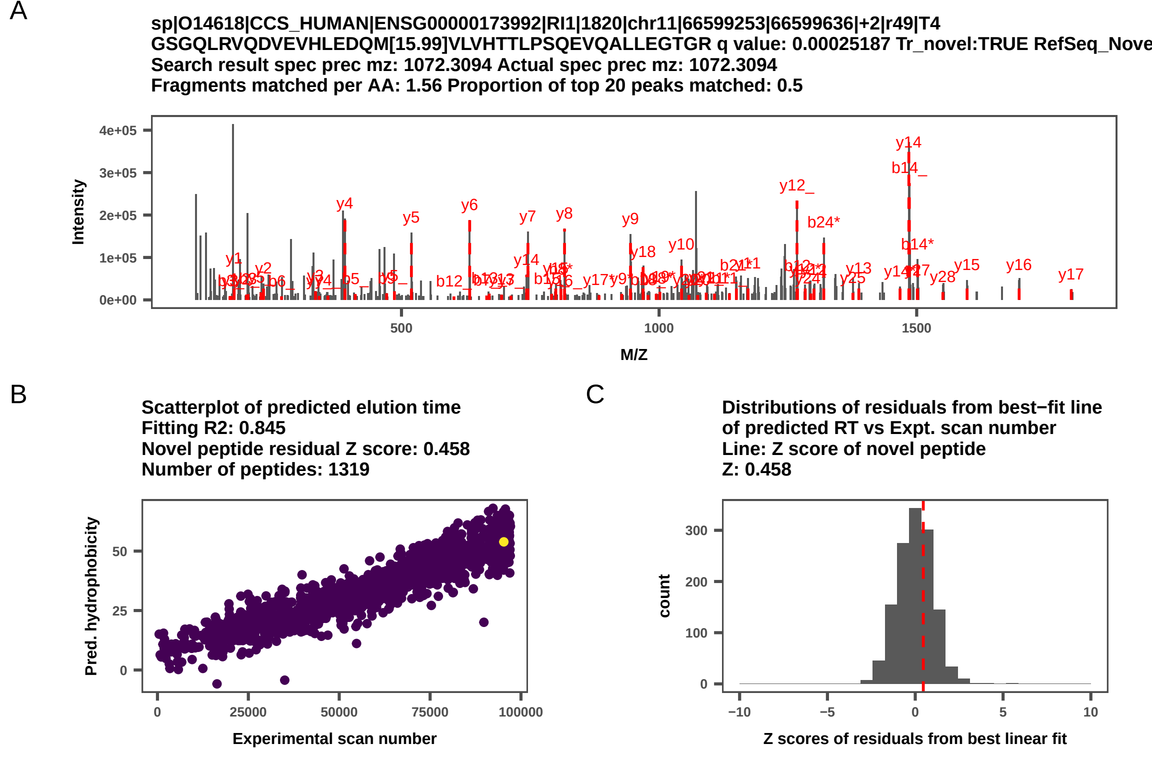
A
sp|O14618|CCS_HUMAN|ENSG00000173992|RI1|1820|chr11|66599253|66599636|+2|r49|T4
GSGQLRVQDVEVHLEDQM[15.99]VLVHTTLPSQEVQALLEGTGR q value: 0.00025187 Tr_novel:TRUE RefSeq_Nove
Search result spec prec mz: 1072.3094 Actual spec prec mz: 1072.3094
Fragments matched per AA: 1.56 Proportion of top 20 peaks matched: 0.5
Intensity
4e+05
3e+05
2e+05
1e+05
0e+00
y14
b14_
y12_
b24*
y4
y5
y6
y7
y8
y9
y10
y18
b14*
y1
y2
b12_
y13
y15
y16
y17
y28
y25
y24
M/Z
500
1000
1500
B
Scatterplot of predicted elution time
Fitting R2: 0.845
Novel peptide residual Z score: 0.458
Number of peptides: 1319
Pred. hydrophobicity
50
25
0
0
25000
50000
75000
100000
Experimental scan number
C
Distributions of residuals from best−fit line
of predicted RT vs Expt. scan number
Line: Z score of novel peptide
Z: 0.458
count
300
200
100
0
−10
−5
0
5
10
Z scores of residuals from best linear fit
